# An open-label extension study to demonstrate long-term safety and efficacy of ABP 501 in patients with rheumatoid arthritis

**DOI:** 10.1186/s13075-019-1857-3

**Published:** 2019-03-29

**Authors:** Stanley Cohen, Jose L. Pablos, Karel Pavelka, Gerard Anton Müller, Alan Matsumoto, Alan Kivitz, Hui Wang, Eswar Krishnan

**Affiliations:** 1 Internal Medicine, Rheumatology Division, Metroplex Clinical Research Center, 8144 Walnut Hill Lane, Suite 800, Dallas, TX 75231 USA; 2 Instituto de Investigación Hospital 12 de Octubre, Madrid, Spain; 3 Institute of Rheumatology, Prague 2, Czech Republic; 4 Abteilung.für Nephrologie und Rheumatologie, Göttingen, Germany; 5 Arthritis and Rheumatism Associates, Wheaton, MD USA; 6 Abteilung für Nephrologie und Rheumatologie, Göttingen, Germany; 7 Biosimilars Development, Amgen Inc., Thousand Oaks, CA USA

**Keywords:** ABP 501, Adalimumab, Rheumatoid arthritis, Long-term safety, Efficacy, Biosimilar

## Abstract

**Background:**

ABP 501 was evaluated in a phase 3 single-arm, open-label extension (OLE) study to collect additional safety and efficacy data in patients with rheumatoid arthritis (RA).

**Methods:**

Subjects completing the final visit in the parent phase 3 randomized, double-blind, controlled equivalence study comparing the efficacy and safety of the biosimilar ABP 501 with adalimumab reference product (RP) were enrolled in this open-label extension (OLE) study. All subjects received 40 mg ABP 501 every other week for 68 weeks. Key safety endpoints included treatment-emergent adverse events (TEAEs), serious adverse events (SAEs), and anti-drug antibody (ADA) incidences. Efficacy endpoints included ACR20 (at least 20% improvement in American College of Rheumatology core set measurements from baseline) and Disease Activity Score 28-joint count C-reactive protein (DAS28-CRP) change from baseline.

**Results:**

Among 466/467 patients treated with ABP 501, 229 transitioned from the ABP 501 arm of the parent study (ABP 501/ABP 501) and 237 from the adalimumab RP arm (RP/ABP 501); 412/467 (88.2%) patients completed the study. The overall TEAE incidence was 63.7% (297/466); grade ≥ 3 TEAE incidence was 9.0% (42/466). The incidence of TEAEs leading to discontinuation of investigational product was 3.6% (17/466). The SAE incidence was 9.9% (46/466). Overall, 18.2% (85/466) of subjects developed binding ADAs and 6.9% (32/466) developed neutralizing ADAs in the OLE study. The ACR20 response rate was 73.3% (340/464 subjects) at OLE baseline, and 78.8% (327/415 subjects) at week 70 of the OLE study. The overall mean DAS28-CRP change from the parent study baseline was − 2.25 at the OLE study baseline (*n* = 440), − 2.36 at week 4 (*n* = 463), − 2.41 at week 24 (*n* = 450), − 2.55 at week 48 (*n* = 433), and − 2.60 at week 70 (*n* = 412). Efficacy was maintained throughout the study.

**Conclusions:**

Efficacy previously demonstrated in the parent study was maintained in this OLE study with no new safety findings. Long-term safety, immunogenicity, and efficacy were similar in the ABP 501/ABP 501 and RP/ABP 501 groups. The single switch from RP to ABP 501 did not impact immunogenicity.

**Trial registration:**

ClinicalTrial.gov, NCT02114931

## Background

Rheumatoid arthritis (RA) is an autoimmune disease characterized by synovial inflammation that results in joint damage. Disease-modifying antirheumatic drugs (DMARDs), such as methotrexate (MTX), modulate the immune system to slow RA progression [1]. Biologics that neutralize tumor necrosis factor (TNF) are the newest DMARDs for which safety and efficacy have been demonstrated, but the use of these agents is cost-prohibitive [2]. Biologics are among the world’s most expensive drugs, accounting for nearly 40% of prescription drug spending in the USA [3]. To lower costs and improve patient access, regulatory agencies have established guidelines for the development of biosimilars, which are biologics that are highly similar to the originator reference product (RP). The expiration of patents on several biologics has spurred interest in biosimilar development [4]. Pharmacoeconomic analyses of biosimilars for RA have suggested that biosimilar use is associated with both significant cost savings and increased patient access to biologics [5, 6].

Unlike small molecule generic drugs, which are identical to their originator agent, biosimilars are large, complex, recombinant proteins and monoclonal antibodies (mAbs) that are highly similar to approved originator biologics. While the exact definition of biosimilars provided by many regulatory agencies varies, the underlying concept of similarity with no clinically meaningful difference is accepted by all, including the European Medicines Agency (EMA), US Food and Drug Administration (FDA), and Japan’s Pharmaceuticals and Medical Devices Agency (PMDA) [7–13]. The regulatory approval process for biosimilars differs from that for originator biologics; for biosimilars, the primary objective is to demonstrate high similarity between the proposed biosimilar and the RP.

Most regulatory agencies have published abbreviated guidelines to support expedited biosimilar development [10–12, 14]. In general, these guidelines outline the following step-wise process: (1) analytical assessment of structural and functional characteristics; (2) preclinical pharmacology assessments; (3) clinical pharmacology, including pharmacokinetic (PK) and pharmacodynamic assessments; and (4) clinical confirmation in at least one representative indication. The purpose of a biosimilar development program is not to evaluate safety and efficacy per se, but to demonstrate similarity to the RP. Phase 2 studies are not needed during biosimilar development; the product moves directly from phase 1 to phase 3 confirmatory evaluations. Phase 3 clinical studies, which are performed in at least one representative indication, compare the efficacy and safety of the biosimilar to the RP [15–18]. The goal of the development program is to follow a totality of evidence approach to demonstrate that the proposed biosimilar is highly similar to the RP. While the European Union (EU) biosimilar market is more well-established with 46 biosimilars authorized for use, the US market is progressing with 12 approvals. The first biosimilar was approved in the USA in 2015 [19, 20].

ABP 501 (EU: AMGEVITA®—adalimumab; USA: AMJEVITA™—adalimumab-atto; Amgen, Inc.) is the first approved biosimilar to the anti-TNF therapy adalimumab (HUMIRA®; Abbvie, Inc.). ABP 501 has been approved in adult patients for the following indications: moderate-to-severe RA, psoriatic arthritis, ankylosing spondylitis (AS), moderate-to-severe Crohn’s disease, moderate-to-severe ulcerative colitis, moderate-to-severe plaque psoriasis, and moderate-to-severe polyarticular juvenile idiopathic arthritis in patients 4 years of age and older. In addition to the approved indications in the USA, additional indications approved in the EU include severe, active, and progressive RA not previously treated with MTX, axial spondyloarthritis without radiographic evidence of AS, uveitis, hidradenitis suppurativa, pediatric Crohn’s disease (≥ 6 years of age), pediatric plaque psoriasis (≥ 4 years of age), pediatric enthesitis-related arthritis (≥ 6 years of age), and polyarticular juvenile idiopathic arthritis (≥ 2 years of age) (EMA AMGEVITA; EMA adalimumab). ABP 501 is a fully human recombinant mAb with the same amino acid sequence as adalimumab RP and was approved as a biosimilar based on the totality of evidence.

Physicochemical similarity of ABP 501 to adalimumab RP was demonstrated by a comprehensive analytical characterization that incorporated orthogonal analytical techniques to assess the identity, general properties, primary and higher-order structure, carbohydrate structure, isoelectric profile, purity, impurities, and thermal-forced degradation profiles as well as the purity, potency, and strength of the mAb product [21]. A comprehensive biofunctional similarity assessment of known mechanisms of action of adalimumab was also completed. Functionally, adalimumab RP and ABP 501 are recombinant IgG1 mAbs that bind to the TNFα cytokine to block its interaction with the p55 and p75 cell surface TNF receptors [22]. Similarity was demonstrated for the following biological properties: kinetics of binding to soluble and transmembrane TNFα, neutralization of TNFα-induced caspase activation and TNFα- and lymphotoxin-α-induced chemokine production and cytotoxicity, binding to relevant Fc-gamma receptors, and effector function activation assessed by antibody-dependent cell-mediated cytotoxicity and complement-mediated cytotoxicity assays with respect to binding to a panel of relevant Fc receptors [22].

ABP 501 was clinically evaluated for PK similarity in a phase 1 study in healthy volunteers. This randomized, single-blind, single-dose, three-arm, parallel-group study demonstrated PK similarity of ABP 501 to adalimumab RP with comparable safety, including a similar incidence of anti-drug antibodies (ADAs). This study was followed by clinical confirmation in two phase 3 studies. The first of these two studies was a randomized, double-blind, active-controlled study in patients with moderate-to-severe plaque psoriasis. In this study, ABP 501 demonstrated similar efficacy and safety to the RP. The primary endpoint, percent improvement in the Psoriasis Area Severity Index (PASI) between baseline and week 16, was 80.9% for ABP 501 and 83.1% for the RP (least-square mean difference of − 2.18 with 95% confidence interval of − 7.39 to 3.02). This study also included a single switch. Subjects with a PASI 50 response at week 16 continued in the study; subjects who had initially been randomized to ABP 501 continued to receive ABP 501, whereas subjects initially randomized to adalimumab were re-randomized (1,1) to continue adalimumab or undergo a single transition to ABP 501. Similar efficacy, safety, and immunogenicity were observed in the transition phase of the study, and a single switch from RP to ABP 501 did not affect these outcomes [23, 24].

The second study was a randomized, double-blind, active-controlled study in patients with moderate-to-severe RA on background therapy with MTX (NCT01970475; referred to herein as the parent study). In this study, similarity between ABP 501 and adalimumab RP was demonstrated based on ACR20 (20% improvement from baseline in the American College of Rheumatology core set of measurements) response at week 24. The ACR20 was 74.6% for ABP 501 and 72.4% for adalimumab RP with a risk ratio (RR) of ACR20 (90% CI) of 1.039 (0.954, 1.133). No new safety signals, clinically meaningful differences in adverse events (AEs), or laboratory abnormalities were observed in the ABP 501 group versus RP [25]. Following this parent study, an open-label extension (OLE) study was conducted to assess the safety of ABP 501 over 72 weeks. We report the results from this open-label, single-arm extension study here.

## Methods

### Study design

**Fig. 1:**
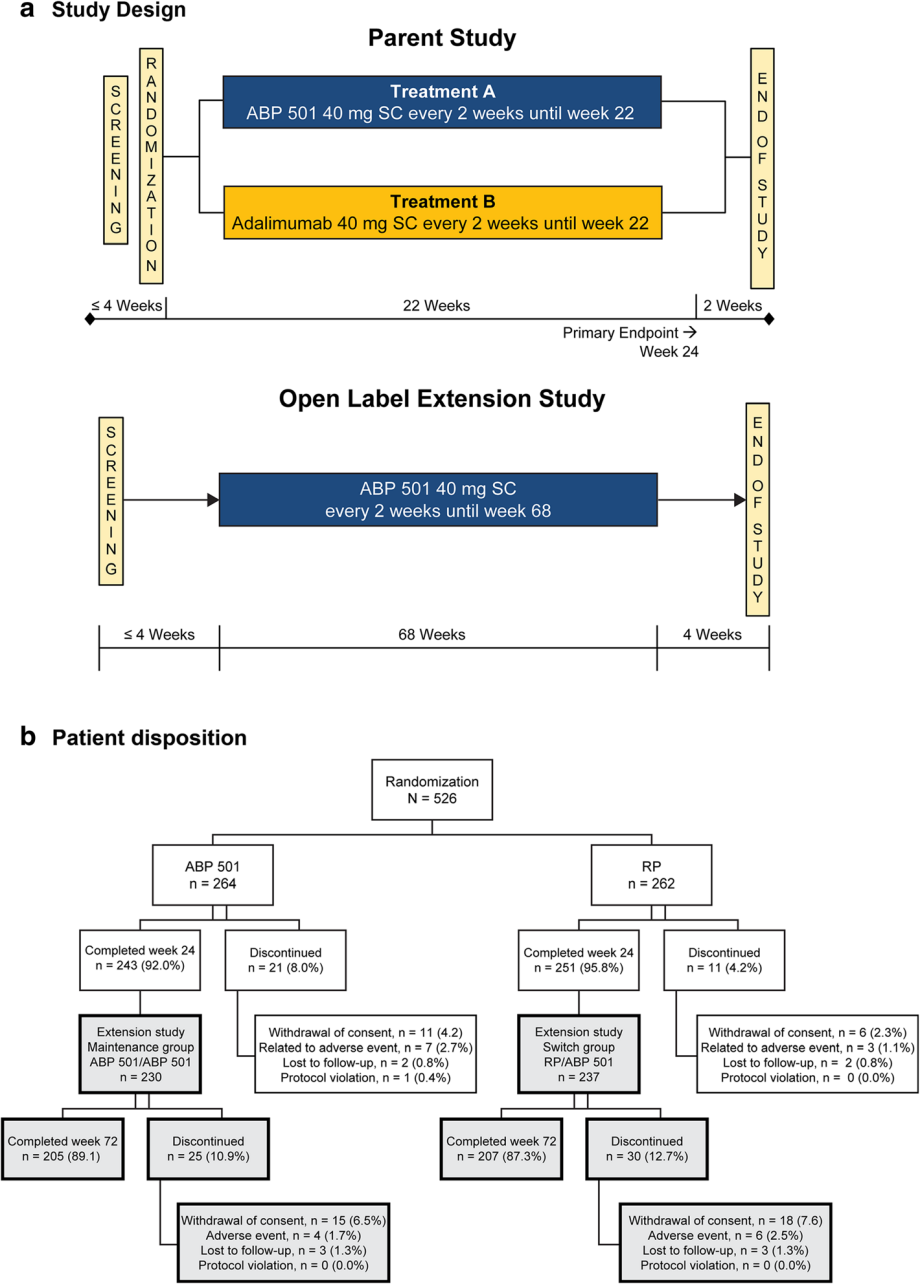
**a** Study design. **b** Patient disposition

This study was an extension of the parent study, which was a randomized, double-blind, active-controlled, 26-week equivalence study in patients with moderate-to-severe active RA despite MTX. Patients in the parent study were randomized 1:1 to receive 40 mg of ABP 501 or adalimumab RP subcutaneously on day 1 and once every 2 weeks through week 22 (Fig. 1a). The primary endpoint was the RR of ACR20 between groups at week 24. The secondary endpoints included Disease Activity Score 28-joint count C-reactive protein (DAS28-CRP), ACR50, ACR70, adverse events (AEs), laboratory evaluations, and ADA measurements. The current study was an open-label, single-arm extension study designed to include 68 weeks of treatment followed by an assessment visit at week 70 and an end of study (EOS) visit at week 72 in immunocompromised RA patients (Fig. 1a). All subjects received ABP 501 40 mg every other week starting on day 1 (the earliest of which was the week 26 visit of the parent study, after the EOS procedures had been completed). Subjects received study drug at the clinic through week 4 and were then allowed to self-dose at home during the weeks in which clinic visits were not required. This study was conducted in 11 countries and 83 centers across Europe and North America.

### Study population

The details of the parent study have previously been published [25]. For inclusion in the present study, subjects were required to have completed the week 26 visit of the parent study. Exclusion criteria included the following: (1) experienced a serious adverse event (SAE) or AE in the parent study that was considered to be potentially detrimental for long-term extension; (2) subject developed an infection requiring the use of oral or IV antibiotics; (3) subject completed the parent study but could not be dosed within 4 weeks of the week 26 visit; (4) subject laboratory values for aspartate aminotransferase, hemoglobin, platelet white blood cell, or estimated creatinine clearance during screening for the OLE study; or (5) subject had developed a significant concurrent medical condition during the parent study.

### Concomitant therapies

Patients were required to receive the same stable dose of MTX for the duration of both the parent study and the extension study, as prescribed by the treating physician. Patients could remain on oral corticosteroids (≤ 10 mg/day of prednisone or equivalent) if on a stable dose for ≥ 4 weeks prior to initiation of the investigational product. Prohibited medications included non-biologic DMARDs (other than MTX) and biologic treatment for RA other than those under investigation [25].

### Safety

Primary outcomes were the incidence (number of patients and number of events) of treatment-related adverse events (TEAEs), SAEs, clinically significant changes in laboratory analytes, and ADA incidence. AEs of interest were also assessed based on the Standard Medical Dictionary for Regulatory Activities queries. AEs and abnormal laboratory results considered as AEs were assigned a toxicity grade according to the National Cancer Institute Common Terminology Criteria for AEs (CTCAE) by the investigator. Immunogenicity, measured by the ADA status, was assessed within 4 weeks of the week 26 visit of the parent study and at weeks 4, 12, 48, and 72 of the extension study. ADA status was assessed using a highly sensitive and drug-tolerant electrochemiluminescent platform as previously described [25].

### Efficacy

Secondary outcome measures included the percentage of patients with an ACR20 response and change in DAS28-CRP between parent study baseline and OLE study baseline and weeks 4, 24, 48, and 70 of the OLE study.

### Statistical methods

For the analysis of this single-arm OLE study, subjects were classified according to their treatment sequence across the parent study and the OLE study; subjects who had transitioned from adalimumab RP in the parent study to ABP 501 in the OLE study are presented as “RP/ABP 501”, and those who remained on ABP 501 across both the parent study and the OLE study are presented as “ABP 501/ABP 501.” All data were summarized descriptively. No inferential analyses were performed.

## Results

Results of the parent study demonstrating similar efficacy and safety between ABP 501 and the RP have previously been published [25].

### Disposition, baseline demographics, and clinical characteristics

In total, 494 (93.9%) subjects completed the parent study. All eligible subjects who completed the week 26 visit of the parent study were offered enrollment in the OLE study. Among these, 467 subjects were enrolled and 466 received at least one dose of investigational product in the OLE study. Among those enrolled, 88.2% completed the study. The most common reason for discontinuation was withdrawal of consent (7.1%). Patient disposition is shown in Fig. 1b.

Patient demographics and baseline characteristics are shown in Table 1. The majority of patients were female (81.2%), white (94.6%), and from Eastern Europe (66%) with a mean age of 55.4 years. Most patients were treatment-naïve for prior use of biologics for RA (72.4%). Concomitant corticosteroid use by patients was limited to ≤ 10 mg prednisone, or equivalent, per day. This included methylprednisolone (29.6%), prednisone (19.1%), and prednisolone (3.6%), which is not considered to be different from the total baseline oral corticosteroid use of approximately 50% as reported in the parent study.

**Table 1:** Demographics and baseline characteristics

Variable	ABP 501/ABP 501 (*N* = 230)	RP/ABP 501 (*N* = 237)	Total* (*N* = 467)^†^
Women, *n* (%)	188 (81.7)	191 (80.6)	379 (81.2)
Age, mean (SD), years	54.7 (11.71)	56.1 (11.40)	55.4 (11.56)
Ethnicity, *n* (%)			
Hispanic or Latino	27 (11.7)	19 (8.0)	46 (9.9)
Not Hispanic or Latino	202 (87.8)	217 (91.6)	419 (89.7)
Race, *n* (%)			
White	218 (94.8)	224 (94.5)	442 (94.6)
Black or African American	8 (3.5)	12 (5.1)	20 (4.3)
Asian	3 (1.3)	0 (0.0)	3 (0.6)
Other	1 (0.4)	1 (0.4)	2 (0.4)
Geographic region, *n* (%)			
Eastern Europe	153 (66.5)	156 (65.8)	309 (66.2)
Western Europe	12 (5.2)	19 (8.0)	31 (6.6)
North America	65 (28.3)	62 (26.2)	127 (27.2)
Duration of RA at baseline of Parent Study, mean (SD), (years)	9.13 (7.873)	9.46 (8.060)	9.30 (7.961)
Duration of RA category at baseline of the parent study, *n* (%)			
≤ 5 years	90 (39.1)	79 (33.3)	169 (36.2)
≥ 5 years	140 (60.9)	158 (66.7)	298 (63.8)
Investigator Global Health Assessment at baseline of the OLE study, mean (SD)	2.5 (1.88)	2.6 (1.80)	2.6 (1.84)
DAS28-CRP at baseline of the OLE study, mean (SD)	3.40 (1.361)	3.32 (1.299)	3.36 (1.329)
Prior biologic use for RA, *n* (%)	60 (26.1)	69 (29.1)	129 (27.6)

*DAS28* Disease Activity Score 28, *RA* rheumatoid arthritis, *RP* reference product, *SD* standard deviation, *ABP 501/ABP 501* patients who continued on ABP 501 from the parent study, *RP/ABP 501* patients who transitioned from RP in the parent study to ABP 501 in the OLE study

*Total = ABP 501/ABP 501 and adalimumab RP/ABP 501 combined because OLE is a single-arm study

^†^Total number of patients enrolled

## Safety

### Treatment-emergent adverse events

Overall, 63.7% of the 466 patients who received at least 1 dose of study drug experienced ≥ 1 TEAE during the extension study of which 9.0% experienced ≥ grade 3 TEAEs (Table 2). The percentages of patients who reported TEAEs were similar in the group that transitioned from adalimumab RP to ABP 501 (65.0%) and the group that continued on ABP 501 (62.4%) from the parent study to the OLE study.

**Table 2:** Overall safety and TEAEs of interest (*n* = 466)*

Variable	Number of patients, *n* (%)
Overall summary of TEAEs	
Any adverse event	297 (63.7)
Any grade ≥ 3 adverse event	42 (9.0)
Any adverse event with the outcome of death	0 (0.0)
Any serious adverse event	46 (9.9)
Treatment-related serious adverse event	3 (0.6)
Any adverse event leading to discontinuation of IP	17 (3.6)
Any adverse event leading to discontinuation from study	8 (1.7)
Most frequent TEAEs by preferred term	
Nasopharyngitis	43 (9.2)
Upper respiratory tract infection	40 (8.6)
Bronchitis	30 (6.4)
Rheumatoid arthritis	29 (6.2)
Hypertension	22 (4.7)
Pharyngitis	19 (4.1)
Serious TEAEs	
Musculoskeletal and connective tissue disorders	12 (2.6)
Infections and infestations	6 (1.3)
Cardiac disorders	5 (1.1)
Eye disorders	4 (0.9)
Gastrointestinal disorders	4 (0.9)
Neoplasms benign, malignant, and unspecified (including cysts and polyps)	4 (0.9)
Reproductive system and breast disorders	3 (0.6)
Vascular disorders	3 (0.6)
Injury, poisoning, and procedural complications	2 (0.4)
Nervous system disorders	2 (0.4)
Surgical and medical procedures	2 (0.4)
General disorders and administration site conditions	1 (0.2)
Hepatobiliary disorders	1 (0.2)
Pregnancy, puerperium, and perinatal conditions	1 (0.2)
Psychiatric disorders	1 (0.2)
Respiratory, thoracic, and mediastinal disorders	1 (0.2)
TEAEs of interest	
Infections	190 (40.8)
Malignancies	8 (1.7)
Hypersensitivity	20 (4.3)
Demyelinating diseases	0 (0.0)
Hematological reactions	5 (1.1)
Heart failure	0 (0.0)
Lupus-like syndrome	0 (0.0)
Liver enzyme elevations	25 (5.4)
Injection site reactions	0 (0.0)

For each category, patients were included only once even if they had multiple events in that category. AEs were coded using MedDRA V.17.1

*AE* adverse event, *IP* investigational product, *TEAE* treatment-emergent adverse event

*One subject did not receive any IP and was not included in these analyses

### Treatment-emergent serious adverse events

A total of 59 SAEs were reported in 46 patients (9.9%) during the study (Table 2). Only 3 patients experienced SAEs that were considered treatment-related. The SAEs reported throughout the study and the number of patients that experienced SAEs were similar in the group that transitioned from adalimumab to ABP 501 (21 events, 8.9% of patients) and the group that continued on ABP 501 (25 events, 10.9% patients). Most of the serious adverse events occurred in one patient. No fatal AEs were reported.

### Treatment-emergent adverse events of interest

TEAEs of interest are reported in Table 2. The most common TEAE of interest was infection, which occurred in 101 (42.6%) patients who transitioned from adalimumab to ABP 501 and 89 (38.9%) patients who continued on ABP 501. The most common infection event identified was nasopharyngitis, which occurred in 25 (10.5%) patients who transitioned from adalimumab to ABP 501 and 18 (7.9%) patients who continued on ABP 501. Other infections included upper respiratory tract infection, bronchitis, pharyngitis, urinary tract infection, sinusitis, oral herpes, tonsillitis, laryngitis, and pneumonia. Demyelinating disease or lupus-like syndromes were not reported during this study.

### Immunogenicity

A total of 466 patients were evaluated for ADAs (binding and neutralizing), and the results are reported in Table 3. Relative to the baseline of the current study, a total of 116 (48.9%) patients who transitioned from adalimumab to ABP 501 and 124 (54.1%) patients who continued on ABP 501 tested positive for binding antibodies at any time during the study; 33 (13.9%) and 33 (14.4%) patients, respectively, tested positive for neutralizing antibodies at any time throughout the study. For subjects with a result at baseline of OLE study, 81 (34.2%) patients who transitioned from adalimumab to ABP 501 and 74 (32.3%) patients who continued on ABP 501 tested positive for pre-existing binding antibodies, and 21 (8.9%) and 13 (5.7%) patients, respectively, tested positive for pre-existing neutralizing antibodies. Relative to the baseline for the parent study, 5 (2.1%) patients who transitioned from adalimumab to ABP 501 and 5 (2.2%) patients who continued on ABP 501 tested positive for pre-existing binding antibodies; no patients tested positive for pre-existing neutralizing antibodies. As expected, the incidence rates of binding and neutralizing ADAs were increased relative to the parent study baseline and were maintained during the OLE study similarly in the group that transitioned from adalimumab to ABP 501 and the group that continued on ABP 501. In the extension study, 18.2% of patients developed binding antibodies relative to the baseline of the OLE, whereas 50.2% of patients developed binding antibodies relative to the baseline of the parent study. In addition, 6.9% of patients developed neutralizing antibodies in the extension study relative to the baseline of the OLE, whereas 14.2% of patients developed neutralizing antibodies relative to the baseline of the parent study.

**Table 3:** Immunogenicity—total antibody developing incidence in OLE study

	Antibody-positive post-baseline of the OLE study with a negative or no result at baseline of the OLE study		Antibody positive in the OLE study with a negative or no result at baseline of the parent study	
	ABP 501/ABP 501 (*N* = 229)	RP/ABP 501 (*N* = 237)	ABP 501/ABP 501 (*N* = 229)	RP/ABP 501 (*N* = 237)
Binding antibody, *n* (%)	50 (21.8)	35 (14.8)	122 (53.3)	112 (47.3)
Neutralizing antibody, *n* (%)	20 (8.7)	12 (5.1)	33 (14.4)	33 (13.9)

*ABP 501/ABP 501* patients who continued on ABP 501 from the parent study, *RP/ABP 501* patients who transitioned from RP in the parent study to ABP 501 in the OLE study

## Efficacy

### ACR20

**Fig. 2:**
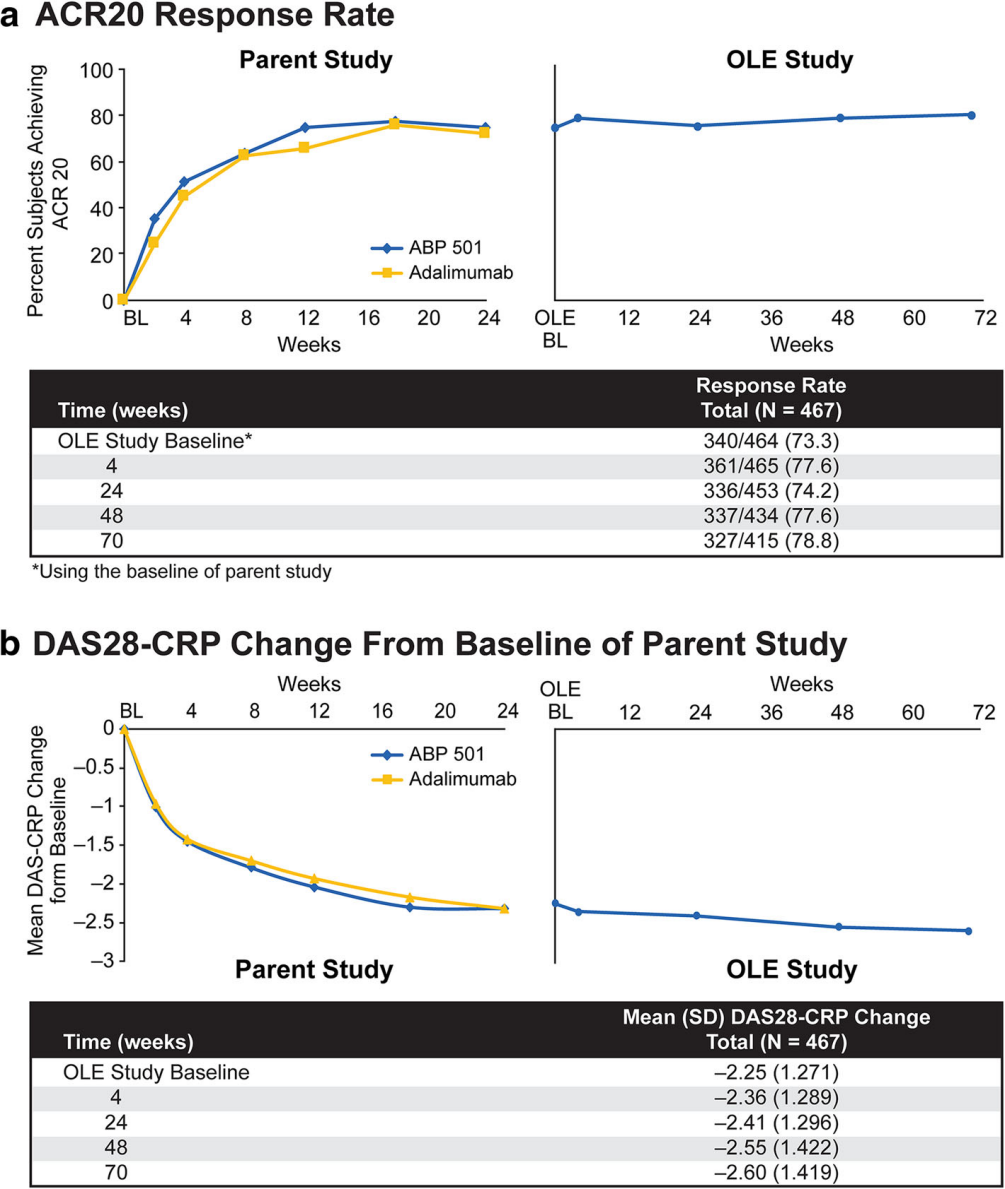
Efficacy: **a** ACR20 response rate; **b** DAS28-CRP change from the baseline of the parent study

The overall ACR20 response rate was 73.3% (340/464) at baseline, and 77.6% (361/465) of the patients met the ACR20 response criteria at 4 weeks (Fig. 2a). At week 24, 74.2% (336/453) of the patients met the ACR20 response criteria; 77.6% (337/434) of patients met the ACR20 response criteria at week 48; and 78.8% (327/415) of patients met the ACR20 response criteria at week 70. The percentages of ACR20 responders were comparable in the group that transitioned from adalimumab to ABP 501 and the group that continued on ABP 501, thus supporting clinical similarity.

### DAS28-CRP

At the baseline of the extension study, the mean change in DAS28-CRP from the baseline of the parent study was − 2.25 (*n* = 440; Fig. 2b). The mean DAS28-CRP change from the baseline of the parent study was − 2.36 (*n* = 463) at week 4, − 2.41 (*n* = 450) at week 24, − 2.55 (*n* = 433) at week 48, and − 2.60 (*n* = 412) at week 70. The percentage of patients who achieved DAS28-CRP remission increased over time in the parent study from weeks 2 to 18 and was relatively unchanged at week 24 and throughout the extension study. Together, efficacy assessed by ACR20 and DAS28-CRP was maintained throughout the period and was similar among the patients who transitioned from adalimumab to ABP 501 and those who continued on ABP 501, thus supporting clinical similarity.

## Discussion

This study provides further evidence of ABP 501 efficacy and safety in patients with RA. In this OLE study, efficacy was maintained and safety was consistent with the known safety profile for adalimumab, regardless of whether patients remained on ABP 501 or transitioned from the RP to ABP 501. No new safety signals were identified. A small number of patients developed binding antibodies in the OLE study; the rates of binding and neutralizing antibodies were similar to those reported in the parent study. ABP 501 was well tolerated during the extension study and displayed an extended safety profile consistent with that of adalimumab. No noticeable differences in the safety profile were observed between the parent study and the OLE study. Similar efficacy was observed among the patients who continued on ABP 501 and those who had transitioned from the RP to ABP 501 based on ACR20 and the change in DAS28-CRP compared to baseline.

ADAs are associated with both unwanted effects and reduced clinical responses to anti-TNF agents. As noted previously, ADA levels in the parent study were higher than those in the pivotal trials of adalimumab in RA; this may be attributed to the use of a more sensitive electrochemiluminescent detection assay and is not unusual relative to other biosimilars. Importantly, the percentage of patients who tested positive for binding and neutralizing antibodies were similar between patients receiving either ABP 501 or adalimumab RP. Once again, in the OLE, the percent of patients who tested positive for binding and neutralizing antibodies was similar between subjects receiving ABP 501 or adalimumab RP. The impact of concomitant MTX, an immune system suppressant, on ADAs is important to consider in a study of anti-TNF therapeutics. In studies of infliximab-treated RA patients, ADA-positive patients who received MTX experienced lower rates of ADA development and improved efficacy compared with those who did not receive MTX [26–29]. Similarly, MTX has been associated with reduced immunogenicity in studies with adalimumab [30]. In both the parent and OLE studies described here, all patients received concomitant MTX which was continued as the same dose throughout the parent and OLE studies. The incidence rates of binding and neutralizing ADAs continued to increase over time during the parent study and were maintained during the OLE as expected based on pivotal trials of adalimumab [31].

The frequency and types of AEs observed here are similar to those reported in studies of TNF inhibitors. One of the most common TEAEs noted in both the parent and OLE studies was the relatively high incidence of nasopharyngitis; however, no difference in incidence was observed between the RP/ABP 501 and ABP 501/ABP 501 dosing groups. Long-term treatment with MTX or the combination of MTX with either the RP or the ABP 501 could have contributed to this outcome.

A strength of this study is the extended safety data for ABP 501 provided over a 72-week period. In combination with the safety data from the parent study, this study provides overall safety information for ABP 501 over a period of 2 years. Limitations of this study include the open-label, single-arm study design, in which bias cannot be eliminated. Bias could have been introduced as a result of patient drop-out over time, especially after 1 year, among those who did not experience therapeutic benefit.

## Conclusions

In concordance with the regulatory requirements for biosimilar development, ABP 501 development began with analytical and nonclinical demonstrations of similarity. PK similarity was then demonstrated in a phase 1 study of healthy volunteers. Finally, clinical confirmation of similarity was achieved in two phase 3 pivotal studies in psoriasis and RA. In conclusion, the results of this open-label, single-arm extension study add to the totality of evidence that demonstrates similarity of ABP 501 to the RP. In addition, transitioning from adalimumab RP to ABP 501 did not impact efficacy, safety, or immunogenicity. This study, combined with the pivotal phase 3 study in patients with RA (the parent study), provides safety results for ABP 501 for a total period of 2 years and further confirms the similarity of ABP 501 to adalimumab RP.

## Abbreviations

ACR20: Response of at least 20% improvement in American College of Rheumatology core set measurements from baseline
ADAs: Anti-drug antibodies
AE: Adverse event
DAS28-CRP: Disease Activity Score 28-joint count C-reactive protein
DMARDs: Disease-modifying antirheumatic drugs
mAbs: Monoclonal antibodies
MTX: Methotrexate
OLE: Open-label extension
PK: Pharmacokinetic
RA: Rheumatoid arthritis
RP: Reference product
SAEs: Serious adverse events
TEAEs: Treatment-emergent adverse events
TNF: Tumor necrosis factor
